# Ocular Surface Changes in Treatment-Naive Thyroid Eye Disease

**DOI:** 10.3390/jcm12093066

**Published:** 2023-04-23

**Authors:** Xulin Liao, Kenneth Ka Hei Lai, Fatema Mohamed Ali Abdulla Aljufairi, Wanxue Chen, Zhichao Hu, Hanson Yiu Man Wong, Ruofan Jia, Yingying Wei, Clement Chee Yung Tham, Chi Pui Pang, Kelvin Kam Lung Chong

**Affiliations:** 1 Department of Ophthalmology and Visual Sciences, The Chinese University of Hong Kong, Hong Kong SAR, China; 2 Department of Ophthalmology, Tung Wah Eastern Hospital, Hong Kong SAR, China; 3 Department of Ophthalmology, Salmaniya Medical Complex, Government Hospitals, Manama 435, Bahrain; 4 Department of Statistics, The Chinese University of Hong Kong, Hong Kong SAR, China

**Keywords:** thyroid eye disease, dry eye, meibomian gland dysfunction, non-invasive tear break-up time, lagophthalmos

## Abstract

Objective: To investigate the association of meibomian gland dysfunction (MGD) and ocular surface exposure with tear film instability in untreated thyroid eye disease (TED) patients. Methods: A cross-sectional study of TED patients from September 2020 to September 2022 was conducted. Ocular surface parameters included ocular surface disease index (OSDI), tear meniscus height (TMH), non-invasive tear break-up time (NITBUT), partial blinking rate, lipid layer thickness (LLT), meibomian gland dropout (meiboscore), Schirmer’s test, and corneal punctate epithelial erosions (PEE). Ocular surface exposure was assessed by the margin reflex distances of the upper and lower eyelid (MRD1 and MRD2), the amount of exophthalmos, lateral flare, and lagophthalmos. Results: In total, 152 eyes from 76 TED patients (64 females and 12 males, age 42.99 ± 12.28 years) and 93 eyes from 61 healthy controls (51 females and 10 males, age 43.52 ± 17.93 years) were examined. Compared with control eyes, TED eyes had higher OSDI, TMH, LLT, and PEE; shorter NITBUT; and worse meiboscore (all *p* < 0.05). They also had larger amounts of exophthalmos, longer MRD1, more lateral flare, and lagophthalmos. Multivariate analysis identified an association of the tear film instability with lagophthalmos (β = −1.13, 95%CI: −2.08, −0.18) and severe MGD in the lower eyelid (β = −5.01, 95%CI = −7.59, −2.43). Conclusions: Dry eye in TED is mainly manifested as evaporative dry eye disease. Severe lower eyelid MGD and worse lagophthalmos were significantly associated with tear film instability in treatment-naive TED patients.

## 1. Introduction

Thyroid eye disease (TED), also known as thyroid-associated orbitopathy or Graves ophthalmopathy, is an autoimmune inflammatory disease related to hyperthyroidism or hypothyroidism. However, some TED patients presented as euthyroid [1,2]. TED patients are usually characterized by deformation, diplopia, visual dysfunction, dry eye, and decreased quality of life. The deformation of TED appearance includes exophthalmos, eyelid retraction, eyelid edema, eyelid redness, and lash ptosis [3,4]. The diplopia of TED is related to inflammation or fibrosis of the orbital contents, especially the extraocular muscle [5]. TED threatens vision, especially in dysthyroid optic neuropathy and exposure keratopathy [6]. TED imposes psychological and economic burdens on patients and affects their quality of life [7]. 

Dry eye symptoms are commonly presented in TED patients [8,9]; some of them have dry eye as the first sign of their ailments. Dry eye disease (DED) is a common eye disease worldwide, affecting around 20% of the population in China [10,11]. According to the Tear Film and Ocular Surface Society Dry Eye Workshop II (TFOS DEWS II) Diagnostic Methodology report, DED can be classified as evaporative and aqueous-deficient dry eye [12]. Evaporative dry eye is characterized by an abnormal lipid layer and is very often caused by meibomian gland dysfunction (MGD). Aqueous deficient dry eye is presented with a low tear meniscus height or Schirmer’s test results. The Asia Dry Eye Society (ADES) added decreased wettability caused by the decrease in membrane-associated mucins as a subtype of the DED [13]. DED can be related with or include inflammation, immune dysregulation, lacrimal gland disorders, and MGD [14]. Low-humidity environments, high body mass index, prostaglandin eye drops, and refractive surgery are also risk factors for DED [15,16]. Immune dysregulation and inflammation involving the eyelid, lacrimal gland, and meibomian glands, as well as excessive ocular surface exposure in TED could be risk factors for DED [17]. Moreover, TED treatment by orbital radiation therapy or steroid pulse has been associated with DED [18,19].

This study aims to analyze the meibomian gland dysfunction, ocular surface exposure, and tear film instability in untreated TED patients with a view to appropriately managing these patients at the early stage of their DED.

## 2. Methods

### 2.1. Study Design and Subjects

This was a cross-sectional study. The TED patients were recruited from the Chinese University of Hong Kong Medical Centre and the Chinese University of Hong Kong Eye Centre from September 2020 to September 2022. This study adhered to the tenets of the Declaration of Helsinki and Ethics approvals (KC/KE-10-0218/ER-3, NTEC Ref. 2010.594) obtained from the Chinese University of Hong Kong. Inclusion criteria included clinical diagnosis of TED [20]. Patients had to be at least 18 years old and had not received treatment for TED, such as orbital radiation therapy or steroid pulse. Patients with incomplete clinical data, history of refractive or other ocular surgery, ocular trauma, and Sjogren’s syndrome were excluded. The healthy controls were age- and sex-matched with the patients, at least 18 years old, and had no underlying ophthalmic diseases. Those who had incomplete clinical data, undergone ophthalmic surgery, or other systemic diseases were excluded. 

### 2.2. Mechanical Ocular Exposure

The margin reflex distance to the upper eyelid (MRD1) is the distance between the upper eyelid margin and the center of the pupillary light reflex in the primary position. The margin reflex distance to the lower eyelid (MRD2) is the distance between the lower eyelid margin to the center of the pupillary light reflex [21]. Exophthalmos was measured by a Hertel exophthalmometer (Keeler Instruments Inc., Broomall, PA, USA). Lagophthalmos is the distance from the upper eyelid margin to the lower eyelid margin when patients close their eyes. Lateral flare is the distance between the upper to lower eyelids just on the lateral side of the corneal limbus [22]. All examinations were conducted by a single senior oculoplastic sub-specialist. 

### 2.3. Dry Eye Assessment

We used the ocular surface disease index (OSDI), which has 12 items and a full score of 100. An OSDI greater than or equal to 13 was regarded as subjective dry eye [23]. The average, maximum, and minimum lipid layer thickness (av-LLT, max-LLT, min-LLT, respectively) and meibography were measured using a Lipiview interferometer (TearScience Inc., Morrisville, NC, USA) [24]. The grading scheme of meibography is the meiboscore (0–3 score for one eyelid) [25]. We regarded 0 and 1 as mild, 2 as moderate, and 3 as severe meibomian gland dysfunction. The tear meniscus height (TMH) and first and average non-invasive tear break-up time (f-NITBUT, av-NITBUT, respectively) were measured by an OCULUS keratograph 5M (Oculus Optikgerate, Wetzer, Germany) [26]. Schirmer’s test (ST-I) without anesthesia was used to test the aqueous tear production. Ocular surface fluorescein staining under a slit lamp was used to evaluate the punctate epithelial erosions (PEE). We applied the Oxford grading scheme (0–6 score) to evaluate the severity of PEE [27].

### 2.4. Statistical Analysis

The continuous variables were presented as mean ± standard deviation. The binary variables were expressed as percentages. The two-group comparison used the Student *t*-test for continuous variables and the Chi-square test for categorical variables. We also conducted univariate and multivariate linear regression to estimate the association of orbital and ocular surface parameters and TED. For the multivariate linear regression model, we adjusted the cofounder, including age, sex, and smoking status. The generalized estimating equation was used to adjust the inter-correlation between two eyes from the same subject. 3 Linear regression models were employed to examine the difference between TED and healthy controls by using orbital and ocular surface parameters as dependent variables. We also applied linear regression models to identify the relationship between the first NITBUT, orbital and ocular surface parameters by utilizing the first NITBUT as the dependent variable. A *p* value less than 0.05 was considered statistically significant. All the statistical analyses were performed using SPSS (IBM SPSS 23.0, SPSS Inc. Armonk, NY, USA) and R-Software R Project https://www.r-project.org/ accessed on 9 March 2023.

## 3. Results

### 3.1. Demographic and Clinical Features of TED Patients and Healthy Controls

We recruited 76 TED patients (152 eyes) and 61 healthy controls (93 eyes) who were age and sex matched (Table 1) Among them, 19.74% TED patients were smokers versus 4.92% of controls. Additionally, 72.37% of TED patients had Graves’ disease. Compared to the controls, the TED patients had larger amounts of exophthalmos, longer MRD1, larger lateral flare, and greater lagophthalmos with statistical significance (Table 2). For the dry eye parameters, the TED patients had higher OSDI, increased TMH, shorter f-NITBUT, shorter av-NITBUT, higher av-LLT, worse meiboscore in both the upper eyelid and lower eyelid, and more severe PEE than the controls with significant differences.

### 3.2. Association of Orbital and Ocular Surface Parameters in TED and Healthy Controls

As shown in Table 3, after adjusting the confounders, including age, sex, and smoking status, the multivariate model showed that TED was associated with exophthalmos, MRD1, lateral flare, lagophthalmos, OSDI, TMH, f-NITBUT, av-NIKBUT, av-LLT, meiboscore in the upper eyelid, meiboscore in the lower eyelid, and PEE. Therefore, the TED patients had more ocular surface exposure, severe objective dry eye symptoms, more unstable tear film, reflex tearing, increased lipid layer thickness, worse evaporative dry eye, and more PEE (Table 2 and Table 3) than healthy controls. Thus, they mainly manifested as having evaporative dry eyes rather than aqueous deficient dry eyes.

### 3.3. Association of Tear Film Instability (f-NITBUT) and Clinical Parameters in TED and Healthy Controls

Tear film instability was associated with the lagophthalmos and severe MGD in the lower eyelid (Table 4). After controlling other factors, for every 1 mm increase in lagophthalmos in TED patients, the non-invasive tear break-up time was shortened by 1.13 s. In comparison to mild MGD in the lower eyelid, the tear break-up time in severe MGD was reduced by 5.01 s. However, the control group did not show any association between the clinical parameters and tear film instability (Table 5).

## 4. Discussion

In this cross-sectional study, we found an association of lower eyelid meibomian gland dropout and lagophthalmos with tear film instability in untreated TED. MGD has a significant impact on the dry eye status of untreated TED patients. Our study found that the influence of MGD on tear film instability is even greater than the impact of mechanical exposure factors.

Treatment-naïve TED patients mainly manifested as having evaporative dry eyes rather than aqueous deficient dry eyes. Many studies have shown that TED patients will have worsened MGD, as Kim et al. reported that TED patients have a higher prevalence of obstructive type MGD [28], and Tugan reported that the meibomian glands are quantitatively decreased in patients with TED [29]. In this study we further proved that the evaporative dry eye rather than the aqueous deficient dry eye affects untreated TED dry eye symptoms. Some studies mentioned that radiation therapy would affect meibomian glands [18] and worsen dry eye [30]. However, other studies reported improvement of the MGD [31] after treatment by steroid pulse and orbital radiation therapies in active thyroid eye disease. 

The tear meniscus height in TED patients was higher than in healthy controls in this study. Both TMH and ST were greater than in healthy controls. A TMH of more than 0.25 mm could indicate that reflex tearing had been reported [32], likely due to the exposure of the eyeball that stimulates the lacrimal glands to secrete tears [33,34]. In some TED patients, the inflammation and swelling could affect the lacrimal gland and disrupt its ability to produce tears [35]. Thus, the relationship between the lacrimal gland and dry eye parameters in TED patients need further investigation.

The MGD in the lower eyelid affects tear film instability in untreated TED. Based on the results of this and other studies, both the upper and lower eyelid meibomian glands are likely to be impaired in TED [36,37]. However, severe deficiency of the lower eyelid meibomian gland significantly impacts tear film stability in TED compared to the upper eyelid MGD. We speculate that this is because the upper eyelid occupies a greater volume and area than the lower eyelid. Thyroid eye disease can cause enlargement of the extraocular muscles or fat, resulting in an increase in the contents of the eye. As a result, when the patient blinks their eyes, a large area of the upper eyelid is squeezed. Therefore, the meibomian gland function of the upper eyelid is slightly better than that of the lower eyelid [38]. Blinking with pressure assists the obstructive meibomian gland to have better secretion. According to the findings of this study, we would recommend gentle compressions and massage during the early stage of TED to produce more relief, especially for the lower eyelid. We also found greater lipid layer thickness in TED than in controls, consistent with reported patients of active TED having more severe MGD but thicker LLT [39]. We think that the TED MGD might be seborrheic MGD [40], with excessive secretion of cloudy meibomian lipids. Moreover, the inflammation of eye contents also affects the meibomian gland and the poor secretion quality of the meibum [41]. Lagophthalmos is incomplete eyelid closure, which results in exposure keratitis [42,43]. Therefore, wearing an eye patch for lagophthalmos during sleep may be beneficial for relieving dry eye symptoms in patients with TED.

Results of this study revealed the relationship between tear film instability, ocular surface exposure, MGD, and other dry eye parameters in untreated TED. There should be attention paid to manage MGD at an early stage in TED. However, this study had the limitation of not evaluating the blinking mechanism and biophysical properties of meibum, which may affect the dry eye status in TED.

In conclusion, treatment-naive TED patients have poor ocular surface status both in objective and subjective dry eye parameters, which mainly manifested as evaporative dry eyes. Lower eyelid MGD and worse lagophthalmos were significantly associated with tear film instability in treatment-naive TED patients. For untreated TED patients, the dry eye status should not be ignored, and the management approach should mainly focus on meibomian gland dysfunction and lagophthalmos.

## Figures and Tables

**Table 1:** Demographics characteristics of thyroid eye disease (TED) and healthy controls.

	TED	Healthy Controls	*p*-Value
Subject numbers	76	61	
Age (years)	42.99 ± 12.28	43.52 ± 17.93	0.836
Onset age (years)	41.26 ± 13.17	NA	
Female:Male	64:12	51:10	0.924
Clinical activity score	0.88 ± 1.19	NA	<0.001
Graves’ disease (N%) ^#^	55 (72.37%)	0 (0.00%)	<0.001
Smoker (N%) ^#^	15 (19.74%)	3 (4.92%)	0.011

^#^ Chi-square test.

**Table 2:** Clinical characteristics of thyroid eye disease (TED) and healthy controls.

	TED	Healthy Controls	*p*-Value
Eye numbers	152	93	
Visual acuity (Log MAR)	0.97 ± 0.16	1.09 ± 0.30	<0.001
**Orbital parameters**			
Exophthalmos (mm)	18.09 ± 2.95	16.29 ± 1.59	<0.001
MRD1 (mm)	5.36 ± 1.63	4.03 ± 0.90	<0.001
MRD2 (mm)	5.03 ± 0.98	5.04 ± 0.55	0.638
Lateral flare (mm)	9.74 ± 2.20	7.23 ± 1.58	<0.001
Lagophthalmos (mm)	0.55 ± 0.88	0.00 ± 0.00	<0.001
**Ocular surface parameters**			
OSDI	28.01 ± 21.70	5.64 ± 3.59	<0.001
Schirmer’s test (mm)	13.67 ± 10.22	12.97 ± 9.57	0.593
TMH (mm)	0.33 ± 0.17	0.26 ± 0.11	<0.001
f-NITBUT (s)	10.16 ± 5.96	15.40 ± 3.71	<0.001
av-NITBUT (s)	15.58 ± 4.71	17.53 ± 2.91	<0.001
av-LLT (nm)	71.03 ± 23.41	62.99 ± 20.53	0.007
max-LLT (nm)	84.84 ± 19.42	79.99 ± 18.74	0.056
min-LLT (nm)	54.64 ± 23.47	50.57 ± 18.92	0.159
Meiboscore upper eyelid	1.64 ± 0.85	1.34 ± 0.63	0.004
Meiboscore lower eyelid	1.36 ± 0.65	1.12 ± 0.69	0.006
Punctate epithelial erosions	0.85 ± 0.73	0.27 ± 0.47	<0.001

Abbreviations: MRD1, margin reflex distance of the upper eyelid; MRD2, margin reflex distance of the lower eyelid; OSDI, ocular surface disease index; TMH, tear meniscus height; f-NITBUT, first non-invasive tear break up time; av-NITBUT, average non-invasive tear break up time; av-LLT, average lipid layer thickness; max-LLT, maximum lipid layer thickness; min-LLT, minimum lipid layer thickness.

**Table 3:** Association of orbital and ocular surface parameters in thyroid eye disease and healthy controls (245 eyes).

	Univariate Model			Multivariate Model *		
	β	95%CI	*p*-Value	β	95%CI	*p*-Value
**Orbital parameters**						
Exophthalmos (mm)	1.80	1.03, 2.57	<0.001	1.77	0.96, 2.59	<0.001
MRD1 (mm)	1.32	0.94, 1.71	<0.001	1.25	0.85, 1.65	<0.001
MRD2 (mm)	−0.01	−0.25, 0.23	0.933	−0.04	−0.28, 0.21	0.768
Lateral flare (mm)	2.52	1.92, 3.12	<0.001	2.55	1.96, 3.14	<0.001
Lagophthalmos (mm)	0.55	0.36, 0.73	<0.001	0.61	0.40, 0.81	<0.001
**Ocular surface parameters**						
OSDI	22.38	17.39, 27.36	<0.001	20.93	16.10, 25.76	<0.001
Schirmer’s test (mm)	0.70	−2.53, 3.94	0.670	0.62	−2.68, 3.91	0.713
TMH (mm)	0.07	0.03, 0.11	0.002	0.07	0.02, 0.12	0.003
f−NITBUT (s)	−5.24	−6.54, −3.94	<0.001	−5.13	−6.51, −3.74	<0.001
av-NITBUT (s)	−1.94	−3.06, −0.83	0.001	−1.84	−2.97, −0.70	0.002
av-LLT (nm)	8.04	1.14, 14.93	0.022	8.99	1.90, 16.09	0.013
max-LLT (nm)	4.85	−1.13, 10.83	0.112	5.56	−0.56, 11.68	0.075
min-LLT (nm)	4.07	−2.44, 10.58	0.221	5.62	−1.12, 12.37	0.102
Meiboscore upper eyelid	0.29	0.05, 0.54	0.017	0.30	0.05, 0.55	0.017
Meiboscore lower eyelid	0.24	0.04, 0.45	0.018	0.21	0.01, 0.42	0.040
Punctate epithelial erosions	0.58	0.40, 0.76	<0.001	0.55	0.37, 0.73	<0.001

* Adjust: Age, Sex, Smoker. Abbreviations: MRD1, margin reflex distance of the upper eyelid; MRD2, margin reflex distance of the lower eyelid; OSDI, ocular surface disease index; TMH, tear meniscus height; f-NITBUT, first non-invasive tear break up time; av-NITBUT, average non-invasive tear break up time; av-LLT, average lipid layer thickness; max-LLT, maximum lipid layer thickness; min-LLT, minimum lipid layer thickness.

**Table 4:** Association of tear film instability and clinical parameters in thyroid eye disease (152 eyes).

	Univariate Model			Multivariate Model *		
	β	95%CI	*p*-Value	β	95%CI	*p*-Value
**Orbit parameters**						
Exophthalmos (mm)	0.10	−0.29, 0.50	0.602	−0.02	−0.37, 0.32	0.898
MRD1 (mm)	−0.33	−0.97, 0.31	0.310	−0.36	−0.96, 0.24	0.243
MRD2 (mm)	0.71	−0.27, 1.69	0.157	0.63	−0.32, 1.57	0.192
Lateral flare (mm)	−0.15	−0.66, 0.35	0.545	−0.25	−0.70, 0.21	0.292
Lagophthalmos (mm)	−0.82	−1.66, 0.03	0.060	−1.13	−2.08, −0.18	0.020
**Ocular surface parameters**						
Schirmer’s test (mm)	−0.01	−0.10, 0.09	0.874	−0.03	−0.12, 0.06	0.490
TMH (mm)	2.55	−3.55, 8.64	0.413	1.25	−4.21, 6.70	0.654
av-LLT (nm)	−0.02	−0.05, 0.02	0.336	−0.01	−0.04, 0.03	0.732
max-LLT (nm)	0.00	−0.04, 0.04	0.840	0.02	−0.03, 0.06	0.451
min-LLT (nm)	−0.02	−0.06, 0.02	0.350	−0.02	−0.05, 0.02	0.400
Punctate epithelial erosions	−1.30	−2.45, −0.16	0.026	−1.08	−2.21, 0.05	0.061
Meiboscore upper eyelid						
Mild	Reference			Reference		
Moderate	0.49	−1.81, 2.79	0.678	1.15	−1.11, 3.40	0.318
Severe	−0.33	−3.40, 2.73	0.830	−0.28	−3.06, 2.50	0.842
Meiboscore lower eyelid						
Mild	Reference			Reference		
Moderate	0.82	−1.56, 3.20	0.498	0.83	−1.36, 3.02	0.459
Severe	−5.21	−7.74, −2.68	<0.001	−5.01	−7.59, −2.43	<0.001

* Adjust: Age, Sex, Smoker. Abbreviations: MRD1, margin reflex distance of the upper eyelid; MRD2, margin reflex distance of the lower eyelid; OSDI, ocular surface disease index; TMH, tear meniscus height; av-NITBUT, average non-invasive tear break up time; av-LLT, average lipid layer thickness; max-LLT, maximum lipid layer thickness; min-LLT, minimum lipid layer thickness.

**Table 5:** Association of tear film instability and clinical parameters in healthy controls (93 eyes).

	Univariate Model			Multivariate Model *		
	β	95%CI	*p*-Value	β	95%CI	*p*-Value
**Orbit parameters**						
Exophthalmos (mm)	−0.01	−0.45, 0.43	0.968	−0.01	−0.46, 0.44	0.962
MRD1 (mm)	0.37	−0.44, 1.18	0.371	0.39	−0.47, 1.24	0.378
MRD2 (mm)	0.67	−0.95, 2.29	0.420	0.67	−0.95, 2.28	0.419
Lateral flare (mm)	0.03	−0.39, 0.45	0.891	0.04	−0.38, 0.46	0.853
**Ocular surface parameters**						
Schirmer’s test (mm)	−0.01	−0.09, 0.08	0.831	−0.01	−0.10, 0.08	0.839
TMH (mm)	−1.79	−8.98, 5.41	0.626	−2.13	−9.40, 5.14	0.567
av-LLT (nm)	−0.02	−0.05, 0.02	0.368	−0.02	−0.06, 0.02	0.336
max-LLT (nm)	−0.02	−0.06, 0.02	0.271	−0.02	−0.06, 0.02	0.259
min-LLT (nm)	0.00	−0.04, 0.04	0.943	0.00	−0.04, 0.04	0.970
Punctate epithelial erosions	−0.76	−2.26, 0.74	0.319	−0.82	−2.48, 0.84	0.335
Meiboscore upper eyelid						
Mild	Reference			Reference		
Moderate	−0.67	−2.50, 1.17	0.476	−1.01	−3.05, 1.03	0.334
Severe	−0.56	−1.99, 0.87	0.443	−0.65	−1.91, 0.61	0.310
Meiboscore lower eyelid						
Mild	Reference			Reference		
Moderate	0.26	−2.11, 2.63	0.831	0.30	−2.55, 3.15	0.838
Severe	0.30	−4.15, 4.74	0.896	0.17	−4.39, 4.72	0.943

* Adjust: Age, Sex, Smoker. Abbreviations: MRD1, margin reflex distance of the upper eyelid; MRD2, margin reflex distance of the lower eyelid; OSDI, ocular surface disease index; TMH, tear meniscus height; av-NITBUT, average non-invasive tear break up time; av-LLT, average lipid layer thickness; max-LLT, maximum lipid layer thickness; min-LLT, minimum lipid layer thickness.

## Data Availability

Not applicable.
